# A Review of Cavitation Erosion on Pumps and Valves in Nuclear Power Plants

**DOI:** 10.3390/ma17051007

**Published:** 2024-02-22

**Authors:** Guiyan Gao, Shusheng Guo, Derui Li

**Affiliations:** System Research Department, China Nuclear Power Technology Research Institute Co., Ltd., Pengfei Road, Shenzhen 518026, China; guoshusheng@cgnpc.com.cn (S.G.); liderui@cgnpc.com.cn (D.L.)

**Keywords:** cavitation erosion, pump, valve, nuclear power

## Abstract

The cavitation erosion failure of pumps or valves induces the low efficiency and reduced service life of nuclear reactors. This paper reviews works regarding the cavitation erosion of pumps and valves in the nuclear power industry and academic research field. The cavitation erosion mechanisms of materials of pumps and valves are related to the microstructure and mechanical properties of the surface layer. The cavitation erosion resistance of austenitic stainless steel can be ten times higher than that of ferritic steel. The cavitation erosion of materials is related to the hardness, toughness, and martensitic transformation capacity. Erosion wear and erosion–corrosion research is also reviewed. Erosion wear is mainly influenced by the hardness of the material surface. Erosion–corrosion behavior is closely connected with the element composition. Measures for improving the cavitation erosion of pumps and valves are summarized in this paper. The cavitation erosion resistance of metallic materials can be enhanced by adding elements and coatings. Adhesion, inclusion content, and residual stress impact the cavitation erosion of materials with coatings.

## 1. Introduction

**Figure 1:**
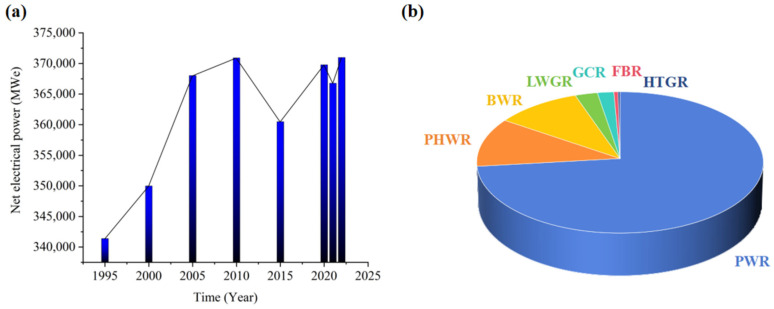
(**a**) Net electrical power of nuclear energy with time; (**b**) number and type of reactors in operation.

The world is faced with a growing electricity demand [1]. Nuclear energy is applied in the electricity industry because of its energy efficiency and reliability; it is especially preferred for areas with limited energy and climate conditions [2,3]. The net electrical power from nuclear energy has been increasing since 1995, as shown in Figure 1. In 2011, the Fukushima accident occurred, impacting global nuclear power development. Nuclear power was replaced by fossil energy in the following years. However, the use of fossil energy to generate electricity has imposed a burden on the environment and natural resources; so, nuclear power development was restored in 2015. Nuclear power has experienced rapid growth in China in recent years [4]. With the development of nuclear technology and science, modern nuclear reactors have become more environmentally friendly and efficient. Pressurized light-water moderated and cooled reactors (PWRs), pressurized heavy-water moderated and cooled reactors (PHWRs), boiling light-water cooled and moderated reactor (BWRs), light-water cooled graphite moderated reactors (LWGRs), and gas-cooled graphite-moderated reactors (GCRs) are first- and second-generation reactors in the industry. Fast breeder reactors (FBRs) and high-temperature gas-cooled reactors (HTGRs) are fourth-generation reactors [5]. Furthermore, fourth-generation reactors, including the sodium-cooled fast reactor, lead-cooled fast reactor, gas-cooled fast reactor, ultra-high temperature gas-cooled reactor, etc., are excellent at nuclear diffusion prevention. Researchers have focused on small modular reactors in recent years because of their natural safety and widespread applicability [6]. However, only a few fourth-generation reactors and small modular reactors are operated, most of which are under conceptual design.

At present, second-generation reactors and third-generation reactors are mainly applied in commercial plants [7]. PWRs are especially preferred in the commercial industry [8,9]. Seventy-three percent of the reactors in operation are PWRs, as shown in Figure 1b. There are mainly two systems in a PWR, the nuclear island system and the conventional island system. The reactor, main pump, steam generator, stabilizer, and their corresponding pipelines and valves are in the primary coolant circuit of the nuclear island. The conventional island system consists of steam turbines, moisture separator reheaters, condensers, pumps, valves, and so on. 

**Figure 2:**
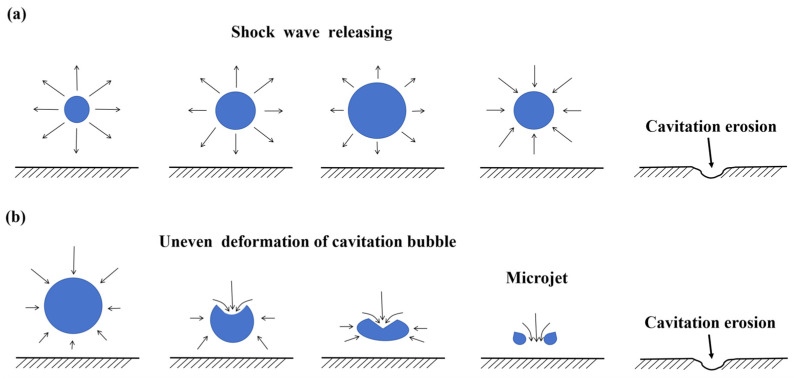
Schematics of (**a**) shock wave mechanism; (**b**) microjet mechanism.

**Figure 3:**
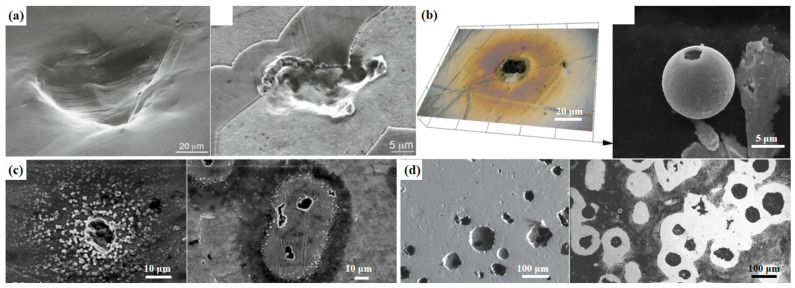
(**a**) Surface deformation pits of austenitic stainless steel and duplex stainless steel [13,27]; (**b**) colorful ring pit and spherical dendritic particle of carbon steel [17,18]; (**c**) ring pits of mild steel in distilled water and tap water [20]; (**d**) disappearance of graphite nodules of nodular cast iron [22] after cavitation erosion.

Cavitation erosion was discovered over one hundred years ago. In the 1950s, researchers realized that cavitation erosion was a kind of damage that was caused by the violent collapses of cavitation bubbles [10,11,12]. Cavitation erosion is common in fluid machinery; during machinery operation, the internal pressure of the equipment fluctuates. Since the 1960s, cavitation erosion has been seen as a result of mechanical effects due to its micro-deformation characteristics and its relationship with mechanical properties. Cavitation bubbles are formed as the local pressure reaches the local saturated vapor pressure. Cavitation bubbles collapse rapidly in high-pressure flow. The stress caused by the cavitation bubble collapse is not in the flow field. Researchers have evaluated that the stress released by the cavitation bubble collapse is able to reach 1 GPa [13]. When the collapses repeatedly occur close to the material surface of the equipment, cavitation erosion can be formed once by high stress and multiple times by low stress. Cavitation erosion is considered as a kind of fatigue; the fatigue strips are shown on the eroded surface. Microjets and shock waves are the dominant mechanisms that are considered the causes of material damage [14,15]. The schematics of the shock wave mechanism and microjet mechanism are shown in Figure 2. Spherical shock waves are released by the cavitation bubble during cavitation bubble growth and compression, as shown in Figure 2a. When the cavitation bubble grows, the pressure inside the cavitation erosion hits the cavitation bubble surface, sending a shock wave inside the bubble and towards the ambient medium of the bubble. The shock wave induces forces on the material surface, causing the cavitation erosion of the material. Microjets are generated by the nonspherical bubble collapse, as shown in Figure 2b. Because the bubble near the solid wall is subjected to uneven pressure, uneven deformation of the bubble occurs. The microjets cause cavitation damage to the material surface. Naudé et al. [14] indicated that the shock wave mechanism greatly influenced cavitation erosion when the collapse took place close to the boundary. It is a complex problem involving unstable fluid flow and material response [16]. In the 1990s, thermal evidence for cavitation was found. The thermal damage characteristics of materials after cavitation erosion further prove that high temperature is released during cavitation bubble collapse. The spherical dendritic particles on the eroded surface of some stainless steel and the iridescent pits on the eroded surface of some mild steel suggest that high temperature occurs during cavitation bubbles collapse [17,18,19]. The ring pits formed in the tap but not formed in the distilled water show that cavitation erosion is caused due to the corrosion effect [20,21]. The dissolution character of the cathodic graphite nodules appearing on the eroded surface of the nodular cast iron with ferrite–pearlite microstructure suggests micro-galvanic activity exists during cavitation erosion [22]. Plastic deformation characteristics such as slip strips are often found on the eroded surface in the incubation period of the cavitation erosion; in addition, craters and micro-cracks are often shown on the eroded surface in the acceleration period [23,24,25,26,27]. The surface topography characteristics of different materials after cavitation erosion are shown in Figure 3. It is generally understood today that cavitation erosion is mainly a kind of mechanical wear in consideration of its damage characteristics. In addition, cavitation erosion interacts with other damage such as corrosion and fretting on the components, and the damage induced by the erosion–corrosion accelerates thinning, causing severe leaks in the nuclear power equipment and resulting in huge investment loss [28]. 

Cavitation erosion is often combined with other damage such as corrosion and wear. Corrosion is an important damage form of structural materials as well. Corrosion destroys the integrity of the equipment. Uniform corrosion is distributed throughout the metal surface, resulting in uniform thinning of the material and reduced structural bearing strength. Local corrosion is concentrated in some areas of the surface, developing to the longitudinal depth. Local corrosion includes pitting corrosion, slot corrosion, intergranular corrosion, and stress corrosion, among which stress corrosion is more harmful [28]. Local corrosion is not easily found, and it is more harmful than uniform corrosion. If the corrosion products in the medium are deposited in the heating part of the circuit, the heat transfer effect will deteriorate, and the scaling part will be damaged due to overheating [29]. Corrosion products are activated into radionuclides under the action of strong radiation fields. Neutron irradiation can be estimated using the trajectory period folding method and the linear chain method [30,31,32]. The protective oxide film induced by corrosion is no longer formed after continuous erosion, resulting in surface damage at a high rate of initial corrosion without protective film. The solid particles in the flow impact the surface, inducing the wear of the component. Cavitation erosion damages the surface material of the component, causing microcracks and microcutting to likely occur, and broadening the damage threshold of the wear. Thus, combined damage is more serious than individual damage separately.

Cavitation erosion and its combination with other surface damage thin the pressure boundary and break the internal structure of the valves and pumps. In this paper, cavitation erosion that has occurred in valves and pumps in nuclear power plants in recent years is reviewed to investigate the mechanism of cavitation erosion and to develop measures for handling it. Test methods for studying cavitation erosion are summarized. Cavitation erosion characteristics of different materials used for valves and pumps are analyzed and summarized. In addition, prevention measurements for cavitation erosion are suggested. Lastly, factors that impact cavitation erosion resistance are summarized.

## 2. Cavitation Erosion Test Methods

A water tunnel, depression tank, venturi, rotating disk, ultrasonic vibration, and cavitating jet have been utilized to study cavitation erosion. A water tunnel can be used to study submarines, torpedoes, submarines, propellers, rudders, hydraulic machinery runners, etc., and the flow rate and pressure can be adjusted independently [12]. A water tunnel is suitable for a wide range of parts and environments; however, the investment cost is high, and the equipment system is huge. A depression tank is used to study hydraulic structures such as the overflow dam face, drain cavern, and baffle block. The flow speed of the venturi can be adjusted, so that the pressure in each part of the tube of the venturi can be changed. The pressure of the tube throat of the venturi can be decompressed, and cavitation occurs when the local pressure of the tube throat falls below the critical pressure. The cavitation erosion mechanism and cavitation erosion resistance of materials can be researched using the venturi.

An ultrasonic vibration test is highly efficient; it can be used to compare the cavitation erosion resistance of different materials and to study the mechanism, damage process, and influence factors of cavitation erosion [33,34]. Cavitation is caused by the high frequency (20 kHz) vibration of the submerged specimen. The vibration tip can be used as the specimen, or the specimen can be fixed under the vibration tip at a small distance (about 0.5 mm). A rotating disk is suitable for generating cavitation and cavitation erosion through specific components in the medium flow process. A rotating disk is often used to study the cavitation erosion in the components such as propellers, impellers, and pipes. The velocity and impact angle are focused on during the tests [35,36]. The sand velocity is enhanced by cavitation bubble collapse, leading to severe damage. A cavitating liquid jet method can be utilized to compare the cavitation erosion resistance of solid materials, and to compare the cavitation erosion ability of various liquids. Cavitation jets with a certain velocity can be produced through various overflow sections, and the jet is ejected from the nozzle, resulting in cavitation erosion of the sample at a certain distance from the nozzle [37,38,39]. At present, the rotating disk device, ultrasonic vibration, and cavitating jet are common methods for cavitation erosion research in the laboratory. The rotating disk method, ultrasonic vibration method, and cavitating liquid jet method are standardized by ASTM G73, ASTM G32, and ASTM G134, respectively [40,41,42].

## 3. Cavitation Erosion on Valves and Pumps

### 3.1. Cavitation Erosion on Valves

Valves are important to control components in fluid transfer systems of nuclear power plants; they have the functions of truncation, regulation, diversion, prevention of counter-current, voltage regulation, shunt, and overflow pressure relief [43,44]. Gate valves, stop valves, check valves, and safety valves are the most widely used valves in nuclear power plants. The valves are distributed in the primary circuit system, the secondary circuit system, the auxiliary system, and the pipeline.

**Figure 4:**
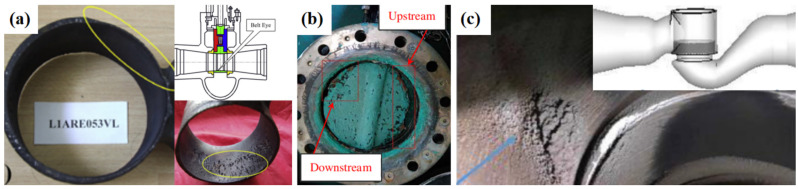
Cavitation erosion of (**a**) the belt eye of a parallel slide valve [45]; (**b**) the sealing surface of the butterfly valve [46]; (**c**) the valve body of the throttle valve [47].

The cavitation erosion of failed valves is shown in Figure 4, the cavitation erosion of the valves occurred in the main feedwater system, circulating water filtration system, and outlet recirculating pipeline of the condensing water system, respectively. The failed parallel slide valve in Figure 4a was located on the bypass pipeline from the steam header to the steam generator. The belt eye of the valve was seriously damaged with thinning and deep macro pits after service for 8 years. The misalignment between the reducer and the belt eye on the upstream side caused the cavitation and induced the cavitation erosion of the downstream belt eye [45]. As shown in Figure 4b, the erosion was found at the upstream head face and downstream head face of the failed butterfly valve. Research showed that the cavitation erosion of the butterfly valve was impacted by the opening angle and inlet pressure [46]. The location of the cavitation erosion, shown in Figure 4c, was at the downstream of the valve body, and the damage was also found near the orifice exit on the valve cage. The control valve is located in the outlet recirculation pipeline of the condensing water system, and it is used to throttle the flow and relieve the pressure. Analysis showed that the opening degree and the length of the downstream divergent connection affected the cavitation erosion of the control valve [47]. To improve the cavitation erosion and prolong the service life of the valve, the structure of the eroded area is suggested to be redesigned and the material of the eroded area is recommended to be replaced by cavitation erosion-resistant material.

Either the collapse of a single cavitation bubble or cavitation bubbles can induce the damage of material solid, which causes the size effect of cavitation erosion. The bubble growth and collapse can be expressed by the Rayleigh–Plesset equation as follows:

(1) $$\frac{p_{B}\left(t\right)-p_{\infty }\left(t\right)}{\rho_{L}}=R\frac{d^{2}R}{dt^{2}}+\frac{3}{2}{\left(\frac{dR}{dt}\right)}^{2}+\frac{4\nu_{L}}{R}\frac{dR}{dt}+\frac{2S}{\rho_{L}R}.$$

*P_B_* is the pressure within the bubble, *P_∞_* is the pressure far from the bubble, *ρ_L_* is the liquid density, *R* is the bubble radius, *ʋ_L_* is the dynamic viscosity, and *S* is the surface tension [48]. The collapse of the cavitation bubble cluster is hard to express, as the dynamics of cavitation bubble movement are very complicated. A large cavitation bubble contains more energy and induces more severe damage than a small cavitation bubble. A small cavitation bubble experiences a faster growth and collapse period than a large cavitation bubble; in addition, small cavitation bubbles may cause damage after several cycles of growth and collapse [49,50]. 

Slight cavitation erosion causes the metal surface to lose brightness and darken, and the metal surfaces gradually become rough and then develop into pitting needle-like holes. The loss of the surface brightness is due to the cavitation pits and indentation [51,52]. Heavy cavitation erosion makes the metal surface very loose or spongy, and when cavitation erosion is severe, it will perforate the parts, and the whole block can even fall off. Then, the device loses balance, causing periodic vibration, making noise, consuming the energy of flow, reducing the working efficiency of the equipment, and even inducing the sudden interruption of operation. In addition, cavitation erosion shortens the maintenance cycle and the service life of the equipment. The flow characteristics are related not only to the valve structure but also to the working conditions [53]. The valve is mainly composed of a valve body, a valve seat, a sealing part, a disc (gate plate or ball), and a valve stem [54]. The valve body and valve seat are pressure-bearing parts that bear the pressure of the medium. The disc is a pressure control part that controls the flow of the medium [55]. When the liquid passes through the low-pressure area of the valves, the liquid velocity increases, and then the static pressure may reach the vaporization pressure of the liquid, resulting in the formation of cavitation bubbles [56]. When the cavitation bubbles are carried by the liquid flow to the area with high static pressure, the bubbles suddenly collapse, and the collapses occur near the valve material, causing damage to the valve. The low-pressure area is caused by the valve structure, such as the area between the body and disc of the incompletely opening butterfly valve and the reducing pressure area of the multistage depressurization valve [57,58]. 

### 3.2. Cavitation Erosion Mechanism of Valve Materials

Valves are manufactured using cast iron, steel, titanium alloy, and nickel alloy in nuclear power equipment. Most valves are made by casting because the structure of most valves is complex. Some of the small gate valves, globe valves, check valves, and some of the large ball valves, are made by forging. Steel is widely used in valves, because corrosion resistance and heat resistance of steel can be obtained by adjusting different elements; in addition, the high hardness and wear resistance of steel can be obtained by heat treatment [59]. Cast iron with hard secondary phases has good erosion–wear resistance. Gray cast iron has been gradually replaced by nodular cast iron in low-pressure valves, as the ductility and microhardness of the nodular cast iron with spherical graphite nodules are better than that of gray cast iron with lamellar graphite [60]. Corrosion-resistant, high-temperature, and low-temperature valves are fabricated by casting or forging using austenitic stainless steel, such as 304 and 316 [61].

**Figure 5:**
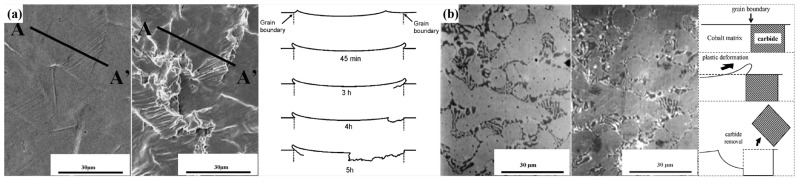
SEM photographs of the eroded surface of (**a**) AISI 304 after cavitation erosion for 0.75 h and 3 h; (**b**) Stellite 6 after cavitation erosion for 0 h and 5 h [70].

Cavitation erosion is closely related to the microstructure because of the size effect of the cavitation erosion [62]. The plastic deformation of the austenitic stainless steel accumulated at the grain boundaries, and then stress concentration was formed, leading to crack initiation. The eroded surface of the austenitic stainless steel is shown in Figure 5a. Stainless steel has shown low resistance to cavitation erosion at the interface. The cavitation erosion of the twin boundaries is lower than other grain boundaries, which is due to the high degree of misorientation of the twin boundaries; the misorientation of grain boundaries can be estimated using the Brandon criterion [63,64]. The hardness and work-hardening capacity impact the cavitation erosion resistance, and the work-hardening capacity is connected with martensitic transformation [65,66,67]. Karimi and Martin found that the cavitation erosion resistance of ferritic stainless steel was lower than that of other stainless steels [68]. 304 austenitic stainless steel has stronger cavitation erosion resistance than BS431S29 martensitic stainless steel due to its martensitic transformation capacity [65,69]. The cavitation erosion of different materials is shown in Table 1. It can be seen that cavitation erosion is closely related to the hardness and microstructure. The cavitation erosion resistance of material with austenite can be ten times higher than that of material with ferrite. The cavitation erosion of austenitic stainless steel is more serious than that of duplex stainless steel. 

### 3.3. Cavitation Erosion of Valve Sealing

The seal of the valve is very important to the valve, which determines the service life of the valve. There are two kinds of sealing materials, soft materials and hard materials. Soft sealing materials include rubber, nylon, fluorine plastic, and so on. Hard sealing materials are metals and alloys, such as copper alloy, chromium stainless steel, and hard alloy. Researchers suggest that cavitation erosion resistance is related to the mechanical properties of the material; the hardness, strain energy, fracture toughness, and fatigue strength correlate with cavitation erosion, and works show that the improvement in the hardness can enhance the wear resistance of the material surface [76,77,78]. Research has shown that microhardness and macrohardness can be utilized to evaluate cavitation erosion resistance [79]. 

The sealing surface of the valve seat is often overlaid by hardfacing alloys such as Co-based Stellite and Ni-based alloys because of their high corrosion resistance and excellent wear resistance [80]. Stellite 6 is often adopted in valves; the sealing surface of the valve is overlaid using conventional welding methods (CWM) such as manual electric arc welding, oxyacetylene flame welding, argon tungsten arc welding, powder isoionization arc welding, and wire plasma arc welding [81]. Stellite 6 is phase transformed by being strain-induced during cavitation erosion, and the microstructure of Stellite 6 is transformed from face-centered cubic (fcc) austenite to close-packed hexagonal (hcp) martensite. With the consideration of the phase transformation theory of Cohen [82,83], shear strain is the main resistance of the phase transformation; the distortion displacement occurs during collapses of cavitation bubbles, resulting in the plastic deformation of Stellite 6. The collapse energy of cavitation bubbles is absorbed by plastic deformation. Stellite 6 is work-hardened during cavitation erosion, and the dislocation density is increased during plastic deformation and work-hardening, leading to the high cavitation erosion resistance of Stellite 6. In one case, plastic deformation accumulated at the interface between the matrix and carbide, cracks were initiated near the carbides, and then the carbides fell off [84]. The eroded surface of Stellite 6 is shown in Figure 5b. 

**Figure 6:**
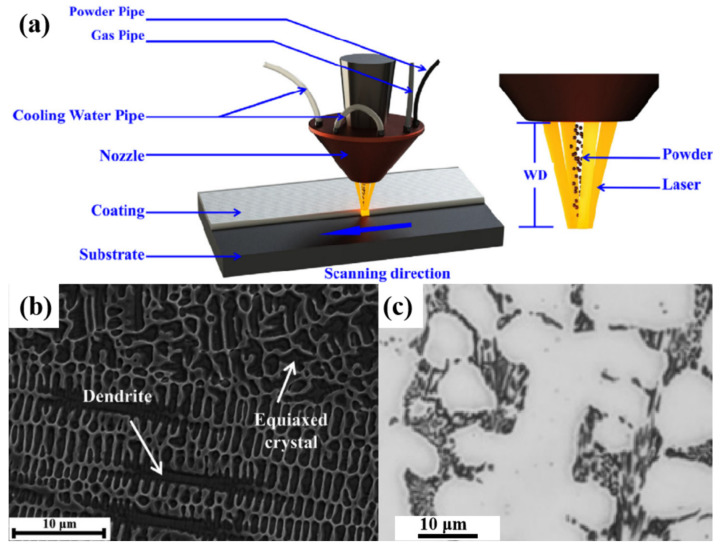
(**a**) Schematic of the laser cladding method (LCM) [89], (**b**) the microstructure of Stellite 6 using LCM [89], and (**c**) the microstructure of Stellite 6 using the conventional welding method [90].

Stellite 6 was not hard enough in the operation, and leaking occurred after some time; thus, researchers have been studying the laser cladding method to give the hardfacing higher hardness [85]. The laser cladding method (LCM) and the microstructure of Stellite 6 are shown in Figure 6. It can be seen that the dendrites of Stellite 6 using LCM are distributed much more evenly in the substrate compared with Stellite 6 using CWM. In addition, the microstructure using LCM is dense and has almost no porosity. The microhardness of Stellite 6 using LCM can reach about 610 HV, while the microhardness of Stellite 6 using CWM is about 450 HV. The surface suffers from microcutting and the impact of abrasive particles under erosion wear. The erosion–wear resistance of Stellite 6 is doubled after LCM treatment. However, LCM is limited by the low efficiency, low accuracy, low deposition thickness, and high dilution rate in the application [86]. The nitrogen ion implantation of Stellite 6 was found to improve the cavitation erosion resistance of Stellite 6 by increasing the content of the γ phase, and the γ phase absorbed energy released by the cavitation load by phase transformation and strengthened the cobalt–solid solution [87,88]. The nitrogen ion implantation seems an effective method to further enhance the cavitation erosion resistance of Stellite 6.

### 3.4. Erosion-Corrosion of Valve Sealing

The damage mechanism of erosion–corrosion is different from cavitation erosion; the electrochemical corrosion characteristics need to be considered. Passive film is formed and broken down repeatedly during the erosion–corrosion process. Cr_2_O_3_ film is formed in a corrosive environment. Stellite alloy in NaOH solution suffers from galvanic corrosion; either new oxides or ions (CrO_4_^2−^) and compounds Co(OH)_3_ are formed after oxide film breakdown [85]. Stellite alloy 706 with an addition of 5 wt.% molybdenum (Mo) and 4.8 wt.% tungsten (W) shows about two times better erosion–corrosion resistance than Stellite 6; however, the carbides in Stellite alloy 706 are higher than those of Stellite 6 [91]. The carbides are Mo-rich carbides and W-rich carbides. Stellite alloys are solid-solution strengthened after adding Mo and W. The stacking fault energy (SFE) is lower than before the addition of Mo and W; then, cross-slip is more difficult, and the work-hardening and the strain to fracture are higher. Stellite alloy 706 shows better resistance to passive film breakdown than Stellite 6 because the corrosive resistant elements such as chromium (Cr) and Mo decrease the charge transfer [92]. 32 wt.% W additions to Stellite 6 increase the solid-particle erosion resistance, which is due to the large size and large number of W-rich carbides [93]. 0.6 wt.% ruthenium additions to Stellite 6 enhance the erosion resistance of Stellite 6 because of their high hardness [94]. Wang et al. [95] suggested that a three-hour heat treatment at a temperature of 1000 °C after spraying could improve the cavitation erosion resistance of the cobalt alloy CoMoCrSi coating twofold, and the improvement was caused by the bonding strengthening of the particle interfaces. They also found that the cavitation erosion mechanisms of CoMoCrSi coating were weak splash adhesion and fragment delamination.

### 3.5. Cavitation Erosion of Promising Valve Sealing Materials

Co-based Stellite alloys may be replaced by Co-free alloys in nuclear power equipment because Co contributes to occupational radiation exposure [96,97]. Co-free alloys such as Fe-based alloys (Fe-Cr-C-Si) have similar cavitation erosion resistance to Co-based Stellite alloys [80]. SFE, twinning, and phase transformation contribute to the good cavitation erosion resistance of the Fe-based alloys with austenitic microstructure. The austenitic phase (λ) is transformed into the martensitic phase (α’ or ε) [98]. Research shows that either 0.3 wt.% boron addition or 10–15 wt.% manganese additions to Fe-based alloys improve the cavitation erosion resistance of Fe-based alloys [99,100]. The improvement is due to the volume fraction reduction in the interfaces between the matrix and carbides. Lin et al. [78,101] found the mass loss of the as-sprayed FeNiCrBSiNbW coating was less than that of the post-annealing FeNiCrBSiNbW coating due to the good fracture toughness, few oxides, and high amorphous content. Co-free alloys such as Ni-based alloy and FeCr-1 alloy show similar erosion–corrosion resistance to Co-based Stellite alloys. The good erosion–corrosion resistance of the Ni-based alloy is attributed to the improved toughness and hardness due to the optimized grain boundaries and intermetallic compounds [102,103]. Fine carbides in the FeCr-1 alloy dispersedly strengthen the matrix, leading to high hardness and erosion–corrosion resistance [104]. The chemical element composition is shown in Table 2.

### 3.6. Cavitation and Cavitation Erosion on Pumps 

Pumps are universal fluid machinery; the centrifugal pump is widely used in nuclear power plants [105]. Pumps are applied in a variety of nuclear power equipment including the main pump, cooling circulating pump, auxiliary feedwater pump, recharging pump, seawater circulating pump, front storage pump, degassing tower draining pump, spent fuel cooling pump, etc. According to the sealing form of the main pump, the main pump is divided into a shaft seal pump and a shield pump. A shaft seal pump is efficient and easy to manufacture and maintain; in addition, it needs low initial investment, and it has a high moment of inertia. So, for large nuclear power plants, the main pump used is generally a shaft seal pump [106]. The flow parts are generally made of austenitic stainless steel. The impeller of the shield pump and the motor rotor are integrated, and it is installed in the same sealed housing, with no radioactive medium leak; the operation of the shield pump is safe and reliable, but the cost of the shield pump is higher than that of an ordinary motor because of the special structure of the motor, and the efficiency of the shield pump is 10~15% lower than that of the ordinary pump [90]. Therefore, the shield pump is generally applied in floating and small modular nuclear power plants. The secondary feedwater pump increases the pressure of the condensate from the secondary deaerator and sends it to the steam generator. The conventional island circulating water pump provides circulating cooling water to the condenser. The impeller and shaft of the circulating water pump are made of stainless steel.

Cavitation is one of the most critical factors affecting the service life and performance of the pump [107]. Cavitation bubbles are formed in the low-pressure area; then, they follow the flow in the direction of the pump exit, collapsing in the high-pressure area [108]. Cavitation erosion is considered during the pump selection, and the net positive suction head available (*NPSHa*) can be expressed by

(2) $$NPSHa=\frac{p_{ab}-p_{v}}{\rho g}.$$

**Figure 7:**
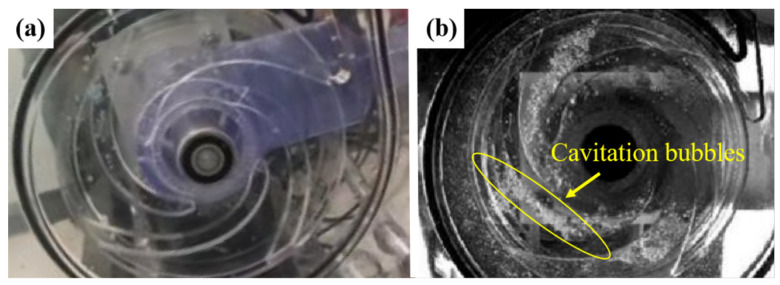
(**a**) Centrifugal pump model; (**b**) cavitation on the pump impeller [115].

*P_ab_* is the absolute pressure, *P_v_* is the vapor pressure of the liquid, *ρ* is the liquid density, and *g* is the gravity acceleration [109]. The net positive suction head required (*NPSHr*) is the minimum pressure of the pump to prevent cavitation. The *NPSHa* must be larger than *NPSHr* [110,111]. However, the *NPSHa* changes with the different working conditions; it tends to be close to the *NPSHr* at a high flow, increasing the cavitation area, and leading to severe cavitation erosion. Cavitation erosion results in the mass loss of the pump impeller, causing resonance and fracture of the pump shaft [112]. Under special conditions such as an accidental power loss and mechanical failure of the main feedwater system, the pump is stopped, and the feedwater pressure is rapidly reduced to below the saturation pressure, resulting in local cavitation formation and unstable cavitation bubbles. When the volume of cavitation bubbles is large, the water column is easily separated, and the pressure rises significantly after the bubbles collapse; then, a water hammer is produced, impacting the equipment, valves, and pipelines, influencing the safe operation of the nuclear power plants [113,114]. In addition, cavitation erosion can be prompted by the high temperature of the working conditions. The impeller is the key flow passage component of a centrifugal pump, and most impellers are made of cast iron, cast steel, and bronze. Cavitation on the pump is shown in Figure 7. 

### 3.7. Cavitation Erosion Mechanism of Pump Materials

**Figure 8:**
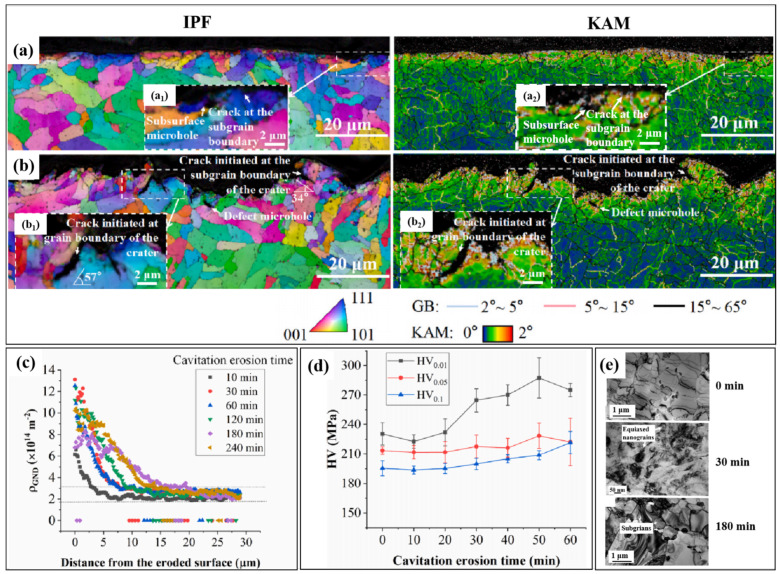
(**a**,**b**) IPF and KAM of cross sections; (**c**) density of GND; (**d**) microhardness; and (**e**) TEM of the surface and subsurface of eroded specimens of martensitic stainless steel after cavitation erosion for different times. (**a_1,2_**,**b_1,2_**) Local magnification images in (**a**,**b**) [118].

In the nuclear power plant, the impeller, shaft, and casing of the feedwater pump are made of martensitic stainless steel. The martensitic stainless steel is usually applied after tempering; the tempering strengthens the matrix by carbides dispersed on the matrix and martensitic laths, resulting in high toughness, wear, and cavitation erosion resistance [116,117]. Inverse pole figures (IPF) and kernel average misorientation (KAM) of cross sections of eroded martensitic stainless steel are shown in Figure 8a,b. The density of the geometric necessary dislocations (*GND*) is shown in Figure 8c. The relationship between the density of the *GND* (*ρ_GND_*) and KAM can be expressed as follows:

(3) $$\rho_{GND}=\frac{2\theta }{\mu b}.$$

*θ* is the KAM, *μ* is the step size, and *b* is the magnitude of the Burgers vector [118]. It can be seen that the eroded surface is plastically deformed, which is related to the KAM increase; subgrain boundaries and nanograin boundaries are formed, grains of the surface and subsurface are refined after cavitation erosion, cracks are initiated at subgrain boundaries and grain boundaries, and refined grains are formed on the surface and subsurface. Nanograins are shown on the surface, and subgrains are shown on the subsurface after cavitation erosion in Figure 8e; grain refinement contributes to the work hardening and high resistance to the cavitation erosion of the surface layer. The microhardness of the surface layer is increased at the incubation period of cavitation erosion because of work hardening; then, the microhardness is decreased during the acceleration period due to the lack of the material integrity. The hardness of the surface layer is shown in Figure 8d. The cavitation erosion resistance of the martensitic stainless steel can be improved by gas nitriding and laser treatment [119]. The hardness and toughness of the laser-treated martensitic stainless steel are better than those of conventional heat-treated martensitic stainless steel because coarse carbides are dissolved during laser treatment, and finer carbides and more retained austenite are distributed in the microstructure [120,121]. The excellent cavitation erosion resistance of nitrided martensitic stainless steel is attributed to the increase in the nitrogen-expanded austenite phases [122]. 

Duplex stainless steel and super duplex stainless steel are increasingly utilized to make impellers; in addition, duplex stainless steel is also used to make wear rings [123,124,125,126]. The duplex stainless steel is used to manufacture the circulating water pumps and feedwater pumps. The microstructure of duplex stainless steel consists of ferrite and austenite. Duplex stainless steel not only has better mechanical properties but also has better stress corrosion resistance, pitting corrosion resistance, wear corrosion resistance, and fatigue corrosion resistance than ordinary austenitic stainless steel [127]. The cavitation erosion of the duplex stainless steel is initiated at the ferrite, and then the ferrite phase is peeled off; meanwhile, the austenite is deformed with slips in the grains [128,129,130]. The more serious cavitation erosion of the ferrite compared to the austenite is caused by the higher strain-rate sensitivity of the ferrite [131,132]. The superior duplex stainless steel with higher yield strength, ultimate tensile strength, and strain hardening rate has better cavitation erosion resistance than ordinary duplex stainless steel, and superior duplex stainless steel tends to replace ordinary duplex stainless steel [133]. 

### 3.8. Cavitation Erosion of Pump Materials with Coatings

**Figure 9:**
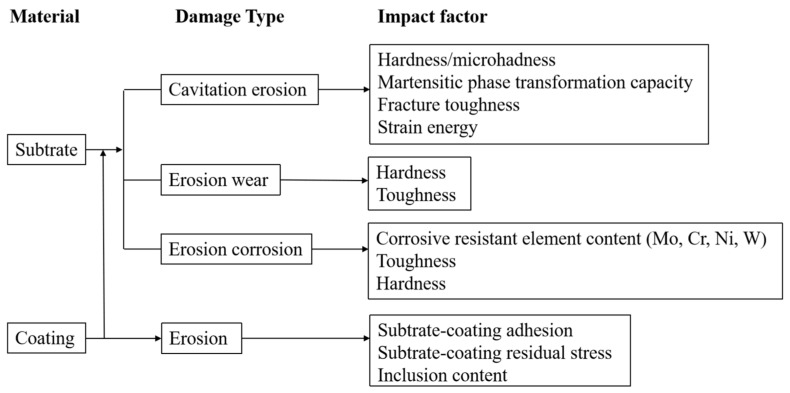
Impact factors of erosion of materials.

In a nuclear power plant overhaul, painting, thermal spraying WC coating, and repair welding are applied to maintain the circulating pumps. However, the maintenance frequency is high, and the effect is general. Hu et al. [134] found the impeller of the circulating seawater pump was seriously eroded by erosion wear, and the impeller with ceramic coating proved to be the optimum coating to resist erosion failure in the operation. The wear-resistant ceramic coating contains high-hard ceramic particles, which can reduce and passivate the sand cutting into the material; in addition, it has good compactness and comprehensive properties [135]. Pędzich et al. [136] found that the cavitation erosion resistance of the ceramic coating was related to the residual stress, and the ceramic coating with compressive residual stress showed better cavitation erosion resistance than that with tensile residual stress. Qiu et al. [137] indicated that the cavitation erosion resistance of the pump coatings was influenced by the coating thickness and adhesion between the matrix and coating. Hong et al. [138] showed that the cavitation erosion resistance of the coating with fewer inclusions between the matrix and coating was better. The coatings on the pumps and valves are listed in Table 3. Wang et al. [139] found that the cavitation erosion resistance of coatings was limited by the adhesion, as the weak metal ceramic bondings were easily peeled off. Mitelea et al. [140] showed that the cavitation erosion resistance of duplex stainless steel could be improved after sensitization at 850 °C for 2 h, which was related to the hardness increase caused by the spinodal decomposition of the ferrite. Bao et al. [141] added Nb to the material surface using the tungsten inert-gas welding powder surfacing method; they found that the Nb-containing duplex stainless steel had a better cavitation erosion resistance than the as-welded duplex stainless steel due to the solid-solution strengthening and twinning-induced plasticity; solid-solution strengthening increased the strength of the surface layer, and twinning-induced plasticity increased the energy absorption of cavitation bubble collapse. Kwok et al. [142,143] showed that laser surface melting (LSM) was beneficial to the enhancement of the cavitation erosion resistance of ordinary stainless steel and that LSM decreased the cavitation erosion resistance of super duplex stainless steel because the ferrite content, which was susceptible to the high strain rate of cavitation erosion, was increased after melting. The impact factors of erosion are summarized in Figure 9. 

## 4. Conclusions

Cavitation erosion that occurred on pumps and valves in nuclear power plants is reviewed in this paper. Works to improve cavitation erosion are widely reviewed and summarized. The conclusions are as follows: 

Damage to pumps and valves is often cavitation erosion combined with solid-particle wear or corrosion, accelerating equipment failure. 

The cavitation erosion resistance of valves can be improved by hardfacing alloys, Co-based Stellite, Ni-based alloys, and Fe-based alloys; in addition, Fe-based alloys are replacing Co-based Stellite in nuclear power plants. Adding elements such as Mo, W, Ru, B, Mn, etc., can enhance the cavitation erosion resistance. 

The cavitation erosion resistance of the pumps can be increased by painting, spraying, and welding, and the ceramic coating shows good cavitation erosion behavior in harsh working conditions. The adhesion, compactness, and inclusion content should be focused on during the coating manufacturing. 

Cavitation erosion is related to the mechanical properties of materials, such as the hardness and toughness. Phase transformation can absorb the energy and decrease the damage caused by cavitation bubble collapse. 

## 5. Future Directions

Cavitation erosion has been studied for more than one hundred years; surface coatings are constantly used to improve the cavitation erosion resistance of the components. Today, the research on the cavitation erosion of pumps and valves should pay attention to the following: (a) the theoretical analysis of the cavitation erosion and the cavitation erosion mechanism should be carefully analyzed and verified through experiments and application, and advanced technologies should be applied to develop an effective surface coating; (b) the medium of the primary and secondary circuits is reflective; so, most connections are welded to prevent the leakage of the radioactive media from causing pollution or causing nuclear safety accidents, and the radiolysis of the coolant and water speeds up the corrosion of the nuclear power equipment. The properties of the equipment materials degenerate under neutron irradiation, becoming brittle and swollen; thus, the cavitation erosion of nuclear materials is severe under the radiolysis environment and neutron irradiation, and this needs to be focused on and studied; (c) the operation conditions of the valves in the nuclear industry are harsh with complex pressure, high temperature, a corrosive environment, and a fluctuating flow rate; it is not rare to see valves fail before their designed lifetime, and a lot of manpower, material resources, and time are spent on maintenance. Thus, monitoring and detecting cavitation erosion methods should be effectively utilized during the operation of nuclear power plants; (d) the research emphasis in the future should be the cavitation erosion resistance of the additively manufactured components and shape memory alloys; in addition, high-entropy alloys seem to be good anti-erosion materials, which can be used to improve the cavitation erosion of the valves and pumps of the nuclear power plants.

## Figures and Tables

**Table 1:** Cavitation erosion of different materials.

Material	Microstructure	Hardness	Mean Erosion Depth (μm)	Mass Loss (mg)	Reference
AISI 420	Ferrite and carbides	210 HV_0.2_	300	-	[71]
AISI 420	Martensite	270 HV_0.2_	180	-	[71]
C45 steel	Ferrite and lamellar pearlite	-	23	-	[23]
EN-GJS-400-15	Ferrite and nodular pearlite	-	15	-	[23]
SS304	Austenite	94	0.21	-	[72]
SS316L	Austenite	84	0.25	-	[72]
Tool steel W1	Martensite	81	0.45	-	[72]
Grey cast iron	Graphite in pearlite	79	1.1	-	[72]
Steel 1.4301	Austenite	270 HV	-	3.5	[73]
Steel 1.4462	Austenite and ferrite	319 HV	-	3.1	[73]
0Cr13Ni5Mo	Martensite	298 HB	-	85	[74]
Cr–Mn–N stainless steel	Austenite–ferrite	240 HB	-	52	[74]
Cr–Mn–N stainless steel	Austenite	211 HB	-	10	[74]
A-NiCrSiB	Ni-based matrix with hard particles	908 HV_0.05_	-	10	[75]
C-NiCrSiB	Ni-based matrix with hard particles	399 HV_0.05_	-	8	[75]
EN-GJL-200	Ferrite, pearlite, and graphite	197 HV_30_	-	156	[75]
X5CrNi18-10	Austenite	209 HV_30_	-	15	[75]

**Table 2:** Chemical element composition of the sealing surface of the valve.

Alloy	Chemical Element Composition											Ref.
	Fe	Cr	C	Si	Co	Ni	B	Mo	W	Mn	Al	
Stellite 21	≤2	27	0.25	-	Bal.	2.5	-	-	-	-	-	[80]
Stellite 3	0.97	30.5	2.4	0.69	Bal.	1.88	1	-	12.5	0.54	-	[85]
Stellite 6	2.09	29	1.25	0.81	Bal.	2.21	-	-	-	-	-	[80]
Stellite 706	1.5	28.6	1.3	-	Bal.	1.6	-	6.1	0.1	-	-	[91]
Stellite 6B	2.8	28.8	1.2	1.3	Bal.	2.1	-	1.5	4.5	1	-	[94]
FeCr-1	Bal.	21	1	2	-	3	2	2	1	3	-	[104]
Fe-based alloy	Bal.	20.11	1.69	0.99	-	-	-	-	-	-	-	[80]
Norem02	Bal.	24.48	1.36	3.32	-	3.88	-	2.02	-	4.47	-	[102]
FeCrCSiB	76.7–77.3	19.8–20	1.7	1.0	-	-	0–0.6	-	-	-	-	[99]
Ni-3	-	24.0–28.0	1.0–1.2	2.4–2.8	-	Bal.	0.8–1.0	4.0–6.0	4.0–5.0	-	6.0–10.0	[102]

**Table 3:** Coatings on the valve and pump.

Coating	Substrate	Coating Process	Damage Resistance Type	Refs.
CoMoCrSi	C22E	Atmospheric plasma spraying	Cavitation erosion	[95]
Stellite 706	Stainless steel	Investment casting or hot isostatic pressing	Erosion–corrosion	[91]
CrCW	Stellite 3/6	Centrifugal casting	Erosion–wear	[93]
Stellite 6	100Cr6	Oxygen fuel spraying	Erosion–wear	[94]
FeCrCSi	AISI 304	Cast ingot	Cavitation erosion	[80]
FeNiCrBSiNbW	1Cr18Ni9Ti	Arc spraying	Cavitation erosion	[78,101]
Ni-based alloy	1Cr18Ni9Ti	Laser cladding	Erosion–corrosion	[102]
Hidroloy 914/AISI 309	Carbon steel	Welding	Cavitation erosion	[67]
Ceramic composite	MAS/6001 25/5	Brushing	Erosion–wear	[134]
YSZ/NiCrBSi composite	AISI 304	Atmospheric plasma spraying	Cavitation erosion	[139]
Silicon carbide and polymer	Stainless steel	Brushing	Cavitation erosion	[137]
Superhydrophobic surface	12X18H10T stainless steel	Nanosecond IR laser treatment	Erosion–wear	[144]
NiAl–Ni3Al intermetallic composites	AISI 420	Laser cladding	Cavitation erosion	[71]
Nanosized TiC ceramic particles	SA 106 Grade B	Liquid metal casting	Cavitation erosion	[59]
WC10Co4Cr	1Cr18Ni9Ti	Oxygen fuel spraying	Erosion–corrosion	[138]

## Data Availability

The data presented in this study are openly available in the publications cited.
